# Supplementary material for: A Critical View of the Application of the APEX Software (Aqueous Photochemistry of Environmentally-Occurring Xenobiotics) to Predict Photoreaction Kinetics in Surface Freshwaters
Source: Molecules. 2019 Dec 18;25(1):9. doi: 10.3390/molecules25010009 (PMC7017383; doi:10.3390/molecules25010009)
Supplement: Supplementary file 1 [file molecules-25-00009-s001.zip › 3.2.4_gcc-4.4.0.zip!/3.2.4_gcc-4.4.0/3.2.4_gcc-4.4.0/doc/octave/html/interpreter/Cell-Arrays.html]

Cell Arrays - Untitled


Next: Comma Separated Lists,
Previous: Data Structures,
Up: Data Containers


---

### 6.2 Cell Arrays

It can be both necessary and convenient to store several variables of
different size or type in one variable. A cell array is a container
class able to do just that. In general cell arrays work just like
N-dimensional arrays with the exception of the use of {
and } as allocation and indexing operators.

- Basic Usage of Cell Arrays- Creating Cell Arrays- Indexing Cell Arrays- Cell Arrays of Strings- Processing Data in Cell Arrays
